# Effect of Propranolol on Motor Cortex Excitability in Essential Tremor: An Exploratory Study

**DOI:** 10.5334/tohm.829

**Published:** 2024-01-02

**Authors:** Adán Miguel-Puga, Gabriel Villafuerte, Mario Treviño, Emmanuel Ortega-Robles, Oscar Arias-Carrión

**Affiliations:** 1 Unidad de Trastornos del Movimiento y Sueño (TMS), Hospital General Dr. Manuel Gea González. Ciudad de México, 14080 México, MX; 2 Laboratorio de Plasticidad Cortical y Aprendizaje Perceptual, Instituto de Neurociencias, Universidad de Guadalajara, Guadalajara, 44130 México, MX

**Keywords:** Essential Tremor, Propranolol, Transcranial Magnetic Stimulation, Cortical Excitability, Noradrenergic Modulation, Physiopathology

## Abstract

**Background:**

Essential tremor, the world’s most prevalent movement disorder, lacks a clear understanding of its pathophysiology. Propranolol, a non-specific beta-blocker capable of crossing the blood-brain barrier, is a primary choice for essential tremor treatment. While its tremor-reducing effects are generally attributed to peripheral actions, various uses hint at central adrenergic effects. Nevertheless, propranolol’s precise impact on the central nervous system in essential tremor subjects remains unexplored.

**Methods:**

In this study, we employed transcranial magnetic stimulation to assess the influence of propranolol on the excitability of the primary motor cortex (M1) in patients with essential tremor, compared to an age- and sex-matched control group. Cortical excitability parameters were measured following placebo and propranolol administration, encompassing resting and active motor thresholds, motor evoked potential characteristics, cortical silent period, and the input/output curve.

**Results:**

Distinct effects were observed across the two cortical hemispheres. Essential tremor patients displayed inhibition of the left M1 cortex and heightened excitability in the right M1 cortex four hours after propranolol administration, but not following placebo.

**Conclusions:**

These findings suggest potential differential noradrenergic excitatory and inhibitory modulation. However, comprehensive understanding necessitates further investigations, including left-handed participants and more diverse essential tremor subpopulations. This study underscores the need for continued exploration to unravel propranolol’s complex effects on motor cortex excitability in essential tremor.

## Introduction

In 1964, Black revolutionized the pharmacology of angina pectoris with the introduction of propranolol [1]. Since then, it has been shown that propranolol, a non-specific beta-adrenergic receptor blocker, has a broader spectrum of action in the body than just a cardiovascular function [2,3]. Its highly lipophilic profile and wide body distribution allows propranolol to move freely across any cell barrier and, therefore, to exert diverse effects in aid of several pathologies, including essential tremor [4].

Essential tremor (ET) is one of the most prevalent movement disorders in the world [5,6,7]. Although this disorder is highly heterogeneous, the most common manifestation is bilateral postural and kinetic tremor affecting the upper extremities [8,9]. Despite first being described more than 130 years ago [10], its physiopathology is still controversial. Most accepted theories point toward a degeneration of Purkinje cells in the cerebellum and subsequent depletion of GABAergic signaling [9]. The wide heterogeneity of ET has led to a recent reclassification of the disease as ‘Essential Tremor Syndrome’ and the addition of an ‘Essential Tremor Plus’ category [11].

Propranolol is a first-line pharmacological treatment for ET, and although it is well known that it crosses the blood-brain barrier [12], its precise effects on the central nervous system have not been fully described. Examples of its uses that involve modulation of the physiology of the central nervous system are the treatment of migraine [13], post-traumatic stress disorder [14], panic attacks [15], anxiety [16,17], schizophrenia [18], autism spectrum disorders [19] and ET [20].

Although advancements in investigating the physiopathology of ET have yielded an enhanced comprehension of the underlying biological mechanisms associated with various drugs targeting the GABAergic system [21], limited research has been dedicated to elucidating the mechanisms underlying drugs like propranolol that operate through alternative pathways. Specifically, the majority of propranolol studies have centered around its efficacy in tremor management, rather than delving into its mode of action. Given that ET is a disorder affecting the central nervous system, it becomes imperative to thoroughly investigate the comprehensive physiological impact of propranolol on the CNS.

An accepted hypothesis of how propranolol produces amelioration of tremor is that proposed by Abila et al. in 1985 [22]. They concluded that its mechanism of action is peripheral and that, contrary to other beta-blockers, the tremolytic activity of propranolol is exclusively via the β2-adrenoceptors in the deep muscle spindles. Nevertheless, this assertion was just a supposition and has not been supported by further experimental evidence. Although their data showed that the Beta-blockers acted peripherally, none of their experiments were intended to ascertain propranolol’s effect in the central nervous system. Therefore, the central effects of propranolol cannot be discarded as a contributor to tremor amelioration.

Evidence from studies on subjects with post-traumatic stress disorder showed that propranolol’s effect on the central nervous system might be modulation of brain activity through the noradrenergic system [23]. Also, neuroimaging studies on subjects with autism spectrum-disorders support the hypothesis that it can increase functional connectivity [24]. However, as β1 and β2 adrenergic receptors have been identified on the cerebral cortex, reticular formation, locus coeruleus, amygdala, hippocampus, striatum, cerebellar cortex and deep nucleus, inferior olive, several thalamus nuclei, hypothalamus, spinal cord, etc. [25,26,27,28], propranolol effects are likely to be broad and widely distributed. In fact, a previous experiment by Baker et al. on healthy subjects showed that propranolol modulated the cortico-muscular coherence on the β band range. While the study by [29] did not reveal any concurrent α-adrenergic modulation, the implications of these findings prompt further investigation into models of disrupted control, such as ET. Their findings provide concrete evidence of propranolol’s impact on motor oscillation control. Since every pathway associated with movement can influence corticospinal tract modulation, our initial investigation centered on the motor cortex. This approach aimed to ascertain whether propranolol induces a central effect in individuals with ET.

Here, we used transcranial magnetic stimulation (TMS) to test the effect of propranolol on motor cortex excitability of subjects with ET. TMS is a safe, non-invasive method to study cortical function that can be used to explore the effects of drugs on different evoked responses of human cortical excitability [30,31]. TMS is based on the Faraday’s principle in which an electrical current running through a coil, creates a magnetic field that goes through the skull and modifies the electrical activity of the underlying neurons. Motor evoked potentials (MEPs) generated by the simple pulse paradigm of TMS are what allow us to test and interrogate the motor cortex [30]. The different variables of MEPs represent an indirect measurement of motor cortex excitability, especially when the input/output (I/O) curve is also calculated, which is a more reliable indicator of cortex excitability [31].

Propranolol’s effects on the motor cortex showed varied patterns in individuals with ET, resulting in specific alterations in motor symptoms and cortical excitability metrics. These findings collectively contribute to a more comprehensive understanding of propranolol’s effects on cortical excitability and its potential implications for essential tremor treatment.

## Methods

### Participants

Between September 2016 and April 2018, two groups were recruited of subjects with an age range of 18 to 75 years. The first group was the ET sample group. A total of 25 subjects were recruited from the patient registry of the Movement Disorders Unit of Hospital General “Dr. Manuel Gea González.” These subjects had been diagnosed recently (<1 year) with ET according to the Movement Disorders Society criteria [8]. Subjects had no history of other brain or movement disorders, major heart conduction disorders, asthma, chronic obstructive pulmonary disease, and they were euthyroid at the time of the study. 17 subjects satisfied these criteria, whereas eight subjects were not included due to cardiovascular or pulmonary disease. One subject quit before the first experimental session because of personal issues unrelated to the experiment or the disease, and another subject was eliminated due to left-handedness. A total of 15 right-handed subjects completed the experiment, and their data were included in the final analysis (10 women, 5 men, mean age 51.86 ± 18.17 years).

For the reference sample group (‘non-ET’ sample), subjects were paired according to age (± 5 years) and gender to the ET sample group. Subjects were right-handed, had no history of brain or movement disorder, major heart conduction disorder, asthma, chronic obstructive pulmonary disease, and were euthyroid. 16 subjects were recruited and satisfied the inclusion/exclusion criteria. 1 subject did not complete the tests because of issues unrelated to the experiment or disease. A total of 15 subjects finished the experiment, and their data were included in the final analysis (10 women, 5 men, mean age 46.13 ± 13.75 years).

All subjects were naive to propranolol and TMS with recent normal electrocardiogram (EKG), thorax radiography, and thyroid functioning tests; also, a current brain magnetic resonance imaging (MRI) excluded cerebrovascular disease and other potential abnormalities. Laterality was assessed by the Edinburgh Handedness Inventory [32]. All subjects provided written informed consent to participate in the present study. The study protocol was reviewed and accepted by the research and ethics committee of General Hospital “Dr. Manuel Gea González,” Mexico (protocol number 49-36-2016) and the research and ethics commissions of the “Facultad de Medicina” of the National Autonomous University of Mexico (UNAM) (protocol number 015/PECEM/2018). The study was conducted following the Declaration of Helsinki. *Supplementary Figure 1* depicts the flowchart of patient selection.

### Experimental Design

We conducted a randomized, double-blind, placebo-controlled crossover study, administering both propranolol and placebo to the essential tremor (ET) sample group and the reference group. Experimental procedures were done in two different sessions. As the half-life of propranolol is about 6 hours [4,33,34], an intersession interval of one week was selected to avoid carryover effects. In each session, four different measures of motor cortex excitability were taken by TMS: a baseline measurement and three consecutive measurements after drug intake, separated by 2 hours (time points were selected by taking into account propranolol’s peak plasma concentration and elimination half-time [4,33,34]). Sessions were carried out at the same time and place for all subjects.

The drug presentation used of propranolol was Inderal® 40 mg by AztraZeneca™, and Splenda™ was used as placebo. Both the propranolol and placebo were encapsulated in hard gelatin capsules to mask the physical appearance of the drugs without altering the pharmacokinetics [35]. After encapsulation, pills were coded (using a random number generation software) and organized in numbered and scheduled pillboxes. A researcher blinded to the pill’s code performed the drug administration. Each pill was given along with 30-40 ml of plain water. All subjects were offered water and food *ad libitum*, and a meal was provided between the 3^rd^ and 4^th^ measurements. As a control to assure blinding, subjects were asked during each session to assess whether they were given the drug or placebo. In the study, only 53% of subjects with essential tremor (ET) and 47% of those in the reference group accurately identified the drug they received during the session. Also, as a positive control of the effect of propranolol, cardiovascular data (resting blood pressure, resting and standing heart rate) was monitored at baseline, 2, 4 and 6 hours after propranolol intake. Additionally, a modified WHIGET tremor scale score was registered at baseline and 4 hours after drug intake (mean peak time of propranolol effect [36]).

### Transcranial Magnetic Stimulation (TMS)

For the single pulse paradigm of TMS, we used a Magstim Rapid^2^ machine (Magstim Co., Whitland Wales UK) connected to a figure-of-eight coil with a wing diameter of 70mm. Measurements were taken immediately after drug administration and subsequently at 2, 4, and 6 hours, covering both cerebral hemispheres and starting with the left hemisphere. To locate and stimulate M1, we employed neuronavigation guided pulses using the Visor2 software and Polaris Vicra 3D camera. Electromyography (EMG) was recorded with Ag-AgCl surface electrodes from the bilateral first dorsal interosseous (FDI) muscles in a belly-tendon montage. EMG was amplified, band-pass filtered 20 Hz–10 kHz and stored for offline analysis.

Subjects were seated in a comfortable chair, and a band with infrared sensors was placed on their forehead. Visor2 software was calibrated using standard MRI guidance. Markers for nasion, right ear, and left ear were located, and at least 40 markers of skull periphery were added. For TMS over M1, the coil was placed tangentially to the scalp with the handle pointing backwards at approximately 45° away from the midline.

The motor hotspot was established as the area on the scalp where single pulses evoked the largest MEP from the FDI muscle in rest. We used the relative frequency method for motor threshold calculation [37]. We defined the resting motor threshold (rMT) as the minimum machine output necessary to achieve a MEP of at least 50 μV in 50% of trials. The active motor threshold (aMT) was determined as the lowest machine output required to elicit a motor evoked potential (MEP) of at least 200 μV in 50% of trials. This was measured while participants pinched at 20% of their maximum voluntary contraction, as gauged by a Baseline® hydraulic pinch gauge 12-0226 (USA).

We recorded 10 resting MEPs at the rMT and 10 MEPs while in contraction at the aMT. MEPs extreme outliers were eliminated from the analysis (<5%). To ensure accuracy, a minimum interval of 6 seconds was maintained between each pulse. For the input/output (I/O) curve measurement, we first recorded 10 motor evoked potentials (MEPs) at the resting motor threshold (rMT) level, maintaining the 6-second pulse separation. We then conducted 10 trials at each of five stimulus intensities, starting from 110% of rMT and increasing in 10% increments up to 150% of rMT. Between each intensity level, participants were given a one-minute rest period to minimize hysteresis effects [38].

As continuous quantitative variables, we recorded the observed rMT, aMT, the amplitude of MEPs (while resting and in contraction), MEPs duration, cortical silent period (CSP) duration, the absolute value of MEPs per intensity on the I/O curve, and the area under the curve. Regarding MEPs amplitude, we report the mean of the individually calculated amplitude. MEPs duration was defined as the time from the beginning of the first deflection to the end of the last deflection. CSP was defined as the time from the end of the last deflection until the return of the EMG activity. MEPs absolute amplitude value of each intensity level of the I/O curve was calculated similarly to MEPs amplitude. The area under the I/O curve was calculated using the trapezoid method for each subject at each time point and treatment.

### Clinical Data and Cardiovascular Data

A researcher, trained before the experiment with a teaching videotape [39], applied the WHIGET tremor scale at baseline and 4 hours after drug intake. This researcher was blinded to drug or placebo administration and made the assessments of all subjects. The scale measures action tremor on 6 test items: postural tremor on 1 position; kinetic tremor on 5 actions: pouring water, drinking water, using a spoon, finger-nose movements, and Archimedes spiral. Every item was graded on a scale from 0 to 4. Points were summarized by subject, left and right hand, to be analyzed as a continuous quantitative variable. A decrease of at least 40% on the WHIGET tremor score was considered to represent a positive response to propranolol administration (this value was calculated at the final analysis after uncovering drug/placebo administration). In the absence of functional MRI, cardiovascular data was monitored and used as a positive control of the effect of Propranolol. Blood pressure and resting heart rate were assessed after at least 30 min at rest. For blood pressure, we used a WelchAllyn® DS44 integrated aneroid and a Littmann® classic II stethoscope. Heart rate was measured with a pulse oximeter (ReliOn™ Model C29, Bentonville, AR). A researcher that was blinded to drug administration carried out all these assessments.

### Statistical Analysis

We conducted a three-way ANOVA to determine the interactions between group (ET vs. non-ET), time (baseline vs. post-treatment), and treatment (placebo vs. propranolol) on the mean values of the WHIGET score, cardiovascular data, and cortical excitability parameters. Additionally, a separate three-way ANOVA was performed for the ET group alone to examine the effects of treatment, time, and brain hemisphere (left or right) on motor cortical excitability variables. To analyze the interactions in the input/output (I/O) curve values, we used a mixed-effects model. For identifying significant differences among the means of the variables of interest, Sidak’s multiple-comparison post hoc test was applied. The normality of data was assessed using the Shapiro-Wilk test, and Bartlett’s test was used to check the homogeneity of variances. Where necessary, the Greenhouse-Geisser correction was applied. We set the threshold for statistical significance at p ≤ 0.05. All figures and statistical analyses were conducted using GraphPad Prism 8 and IBM SPSS® Statistics 21.

## Results

Demographic characteristics of both the essential tremor (ET) and reference group are presented in Table 1. Mean age did not differ between the groups (p = 0.325), with a balanced gender distribution; however, the proportion of females within each group was 2/3.

**Table 1:** Demographic data of Essential Tremor and reference sample groups.


DEMOGRAPHIC DATA	ET SAMPLE (N = 15)	NON-ET SAMPLE (N = 15)

Age (years ± SD)	51.86 ± 17.17	46.13 ± 13.75

Sex F(M)	10(5)	10(5)

Right-handed laterality (n)	15	15

Smokers (n)	2	2

Consumption of CNS acting drugs (n)	0	0

Evidence of neurologic disease other than essential tremor (n)	0	0

Adverse effects (n)	3	1

Mild	3	1

Moderate	0	0

Severe	0	0

Correctly guessed pill content (n)	8	7

Familiar history of essential tremor (n)	7	0

Age at ET diagnosis (years ± SD).	50.8 ± 17.72	NA

Time with disease (years ± SD).	10.08 ± 7.27	NA

History of favorable response to alcohol intake (n)	4	NA


n, number of subjects; SD, standard deviation; F, female; M, male; CNS, central nervous system; ET, essential tremor.

**Figure 1:**
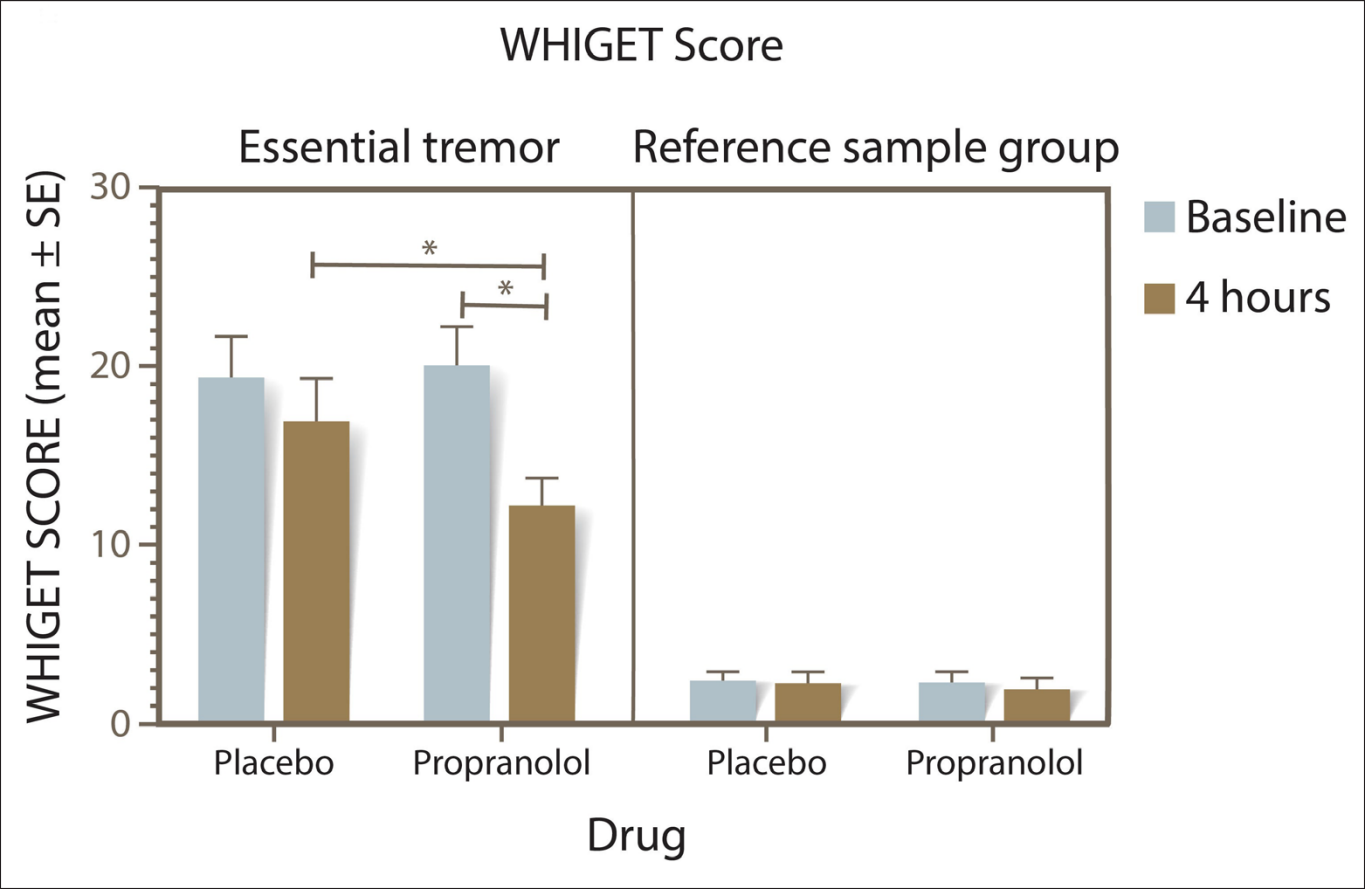
WHIGET tremor score for the ET and reference sample groups after placebo or propranolol intake. *p < 0.05 for the subjects administered propranolol.

Motor symptoms, as shown in Figure 1, revealed a significant three-way interaction between group, treatment, and time (F_1,56_ = 5.540, p = 0.0221). Among the two-way interactions, the interaction of treatment and time was notably significant (F_1,56_ = 6.743, p = 0.0120), aligning with our expectations. Additionally, both treatment (F_1,56_ = 4.328, p = 0.0421) and group (F_1,56_ = 115.9, p < 0.0001) demonstrated significant simple main effects independently. Notably, the ET group showed a marked improvement in tremor score 4 hours after taking propranolol (mean of 7.9, 95% CI 1.7 – 14.1, p = 0.0035), which was not observed with placebo. At baseline, there was no significant difference in tremor scores between placebo and propranolol within the ET group. However, differences emerged at the 4-hour mark (p < 0.001).

Cortical excitability parameters in subjects with ET exhibited distinct patterns. In the left hemisphere of ET subjects, there was a significant three-way interaction between group, treatment, and time factors (F_3,168_ = 2.708, p = 0.0469) in relation to the resting motor threshold (rMT). However, neither the two-way interactions nor the simple main effects reached statistical significance. Similarly, no significant interactions were found among these three factors for the active motor threshold (aMT) or for motor thresholds in the right hemisphere. This pattern was also observed when analyzing the effects of treatment, time, and hemisphere on the rMT and aMT specifically within the ET group.

**Figure 2:**
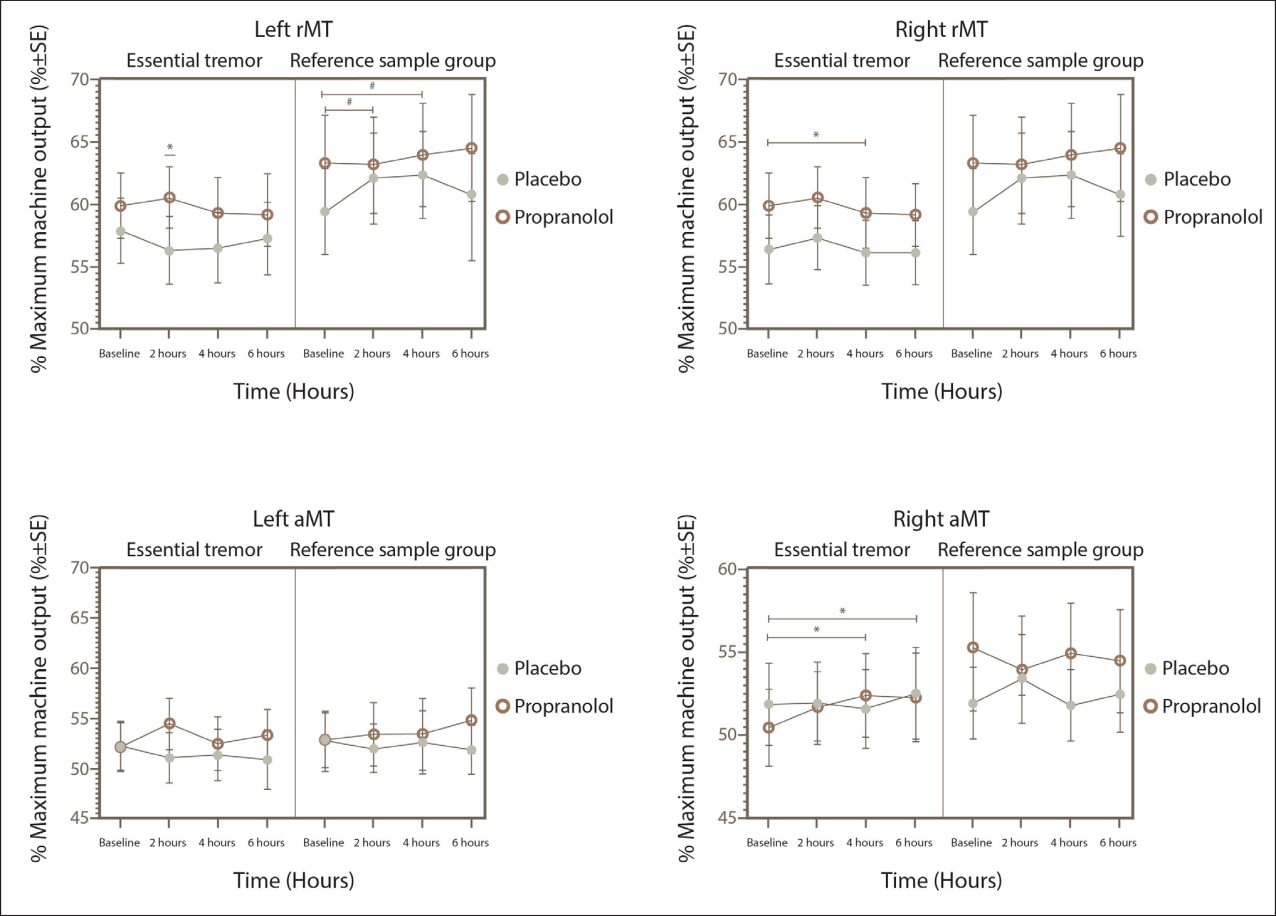
Resting and active motor threshold for the ET and reference sample groups in the right and left hemispheres after placebo or propranolol intake. *p < 0.05 for the subjects given propranolol (rMT, resting motor threshold; aMT, active motor threshold).

Despite the lack of significant interactions, post hoc multiple comparison tests revealed specific changes. After propranolol intake, rMT and aMT in the right hemisphere increased at 4 and 6 hours compared to baseline in the ET group (p = 0.015 for aMT and p = 0.04 for rMT at 4 hours), a change not observed with placebo. Additionally, at 2 hours post-treatment, ET subjects showed an increased rMT in the left hemisphere when administered propranolol (p = 0.013). In contrast, subjects without ET who received propranolol did not exhibit these changes (as illustrated in Figure 2). However, for non-ET subjects receiving placebo, there was a noticeable increase in rMT in the left hemisphere at 2 and 4 hours post-administration compared to baseline (p = 0.017 and p = 0.003, respectively).

MEPs duration remained consistent, showing no significant variations between ET and healthy subjects, or between propranolol and placebo treatments. There were also no observed interactions among these factors. However, in the non-ET group, the cortical silent period in the right hemisphere was notably prolonged at 4 and 6 hours post-baseline (p = 0.026 and p = 0.022, respectively).

**Figure 3:**
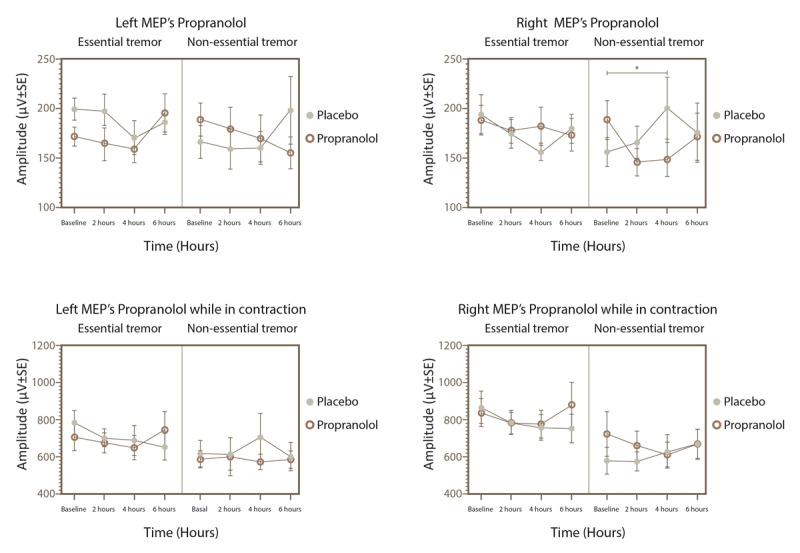
MEPs amplitude and MEPs amplitude while in contraction for the ET and reference sample groups in the right and left hemispheres after placebo or propranolol intake. *p < 0.05 for the subjects given propranolol (MEP, motor evoked potential).

In assessing the amplitude of evoked potentials at 100% of rMT or aMT, no significant interactions were found among the factors, except for a notable group effect on amplitude during muscle contraction (F_1,56_ = 6.081, p = 0.0168). Additionally, no significant interactions emerged in the analysis within the ET group when considering hemisphere as a factor. However, post hoc tests revealed that in the reference group, the amplitude of Motor Evoked Potentials (MEPs) in the right hemisphere, with muscles at rest, decreased at 4 hours post-baseline under the effect of propranolol (p = 0.027). This decrease was not observed in the ET group under any experimental condition. Similarly, MEPs amplitude during muscle contraction did not show any changes (as illustrated in Figure 3).

**Figure 4:**
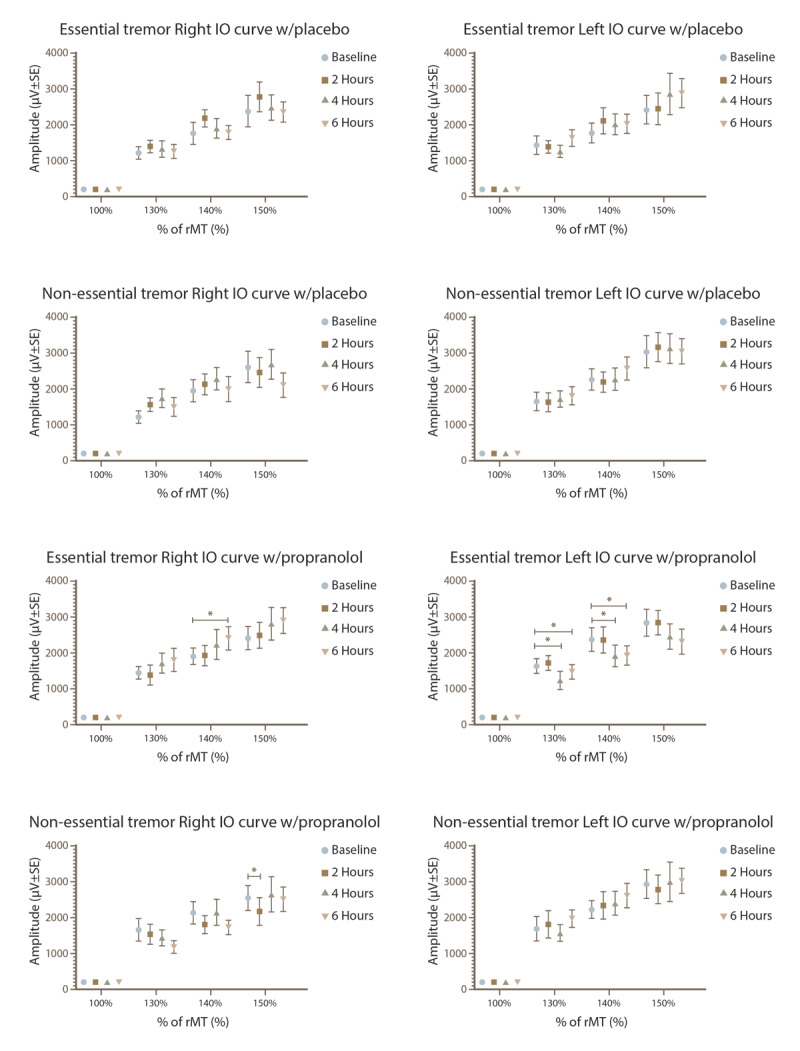
I/O curve in the right and left hemisphere of the ET and reference sample groups after placebo or propranolol intake. *p < 0.05 for the subjects given propranolol.

**Figure 5:**
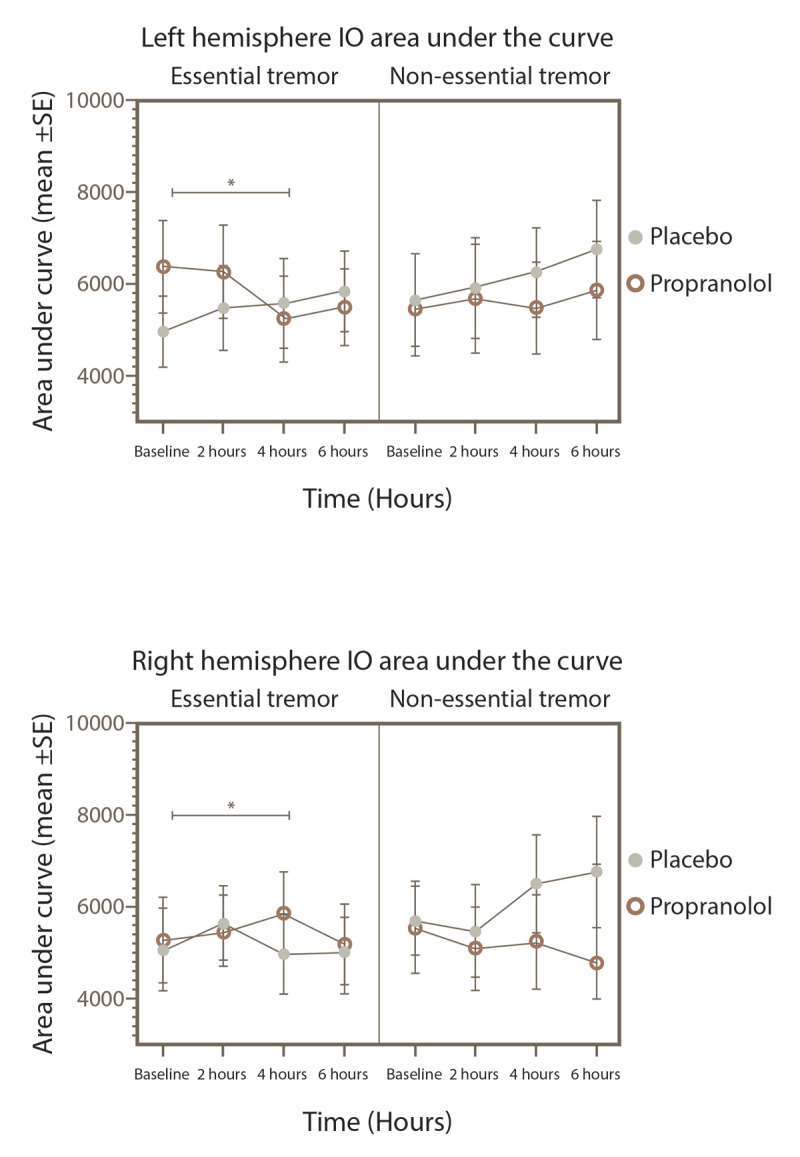
I/O area under the curve in the right and left hemisphere of the ET and reference sample groups after placebo or propranolol intake. *p < 0.05 for the subjects given propranolol.

Significant changes in the I/O curve were observed for both hemispheres, as depicted in Figures 4 and 5. When using mixed-effects models, no interactions between factors were found in the right hemisphere for either the ET group or the reference group. However, subsequent post hoc analysis showed that in ET subjects who received propranolol, there was a notable increase in MEPs amplitude at 140% of the rMT compared to baseline, particularly evident at 6 hours (p = 0.02). In contrast, the reference group exhibited a reduction in MEPs amplitude at 150% of rMT at 2 hours (p = 0.04). No significant amplitude effects were observed with placebo administration in either group.

In the left hemisphere, our analysis revealed no significant interactions among the factors. However, within the essential tremor (ET) group treated with propranolol, a significant main effect of time was observed (F_2.618, 36.66_ = 3.761, p = 0.0231). Following multiple comparisons analysis, it was found that the MEPs amplitude decreased after propranolol administration at 4 and 6 hours compared to baseline, specifically at intensities of 130% and 140% of the rMT (p < 0.05 in all cases). At 150% intensity, no significant differences were observed. On the other hand, subjects in any group who received placebo did not show any changes in MEP amplitude across all stimulus intensities.

The analysis of variance revealed no interactions between factors for the area under the curve in either hemisphere. However, post hoc analysis notably showed a reduction at 4 hours compared to baseline in subjects with essential tremor (ET) who were administered propranolol, affecting both hemispheres (p = 0.036). No such changes were observed in those given the placebo. Additionally, the reference group did not exhibit any amplitude changes at any point in the study.

Cardiovascular data from the reference group, as shown in *Supplementary* Figure 2, demonstrated that both systolic and diastolic blood pressure decreased in subjects administered propranolol at 2 and 4 hours post-baseline, aligning with expectations [34]. A significant interaction was observed between time and treatment for heart rate when subjects were treated with propranolol (F_3, 168_ = 7.317, p = 0.0001). Additionally, significant main effects of time were noted for both systolic and diastolic blood pressures, as well as for heart rate. In contrast, subjects who received placebo did not show any decrease in these cardiovascular measures.

## Discussion

Our findings unveil a distinctive influence of propranolol on the left and right hemispheres, highlighting a disparity in the response to the drug between individuals with ET and those without the condition.

At the 2-hour mark following propranolol administration, a subtle reduction in excitability was evident in the right motor cortex among participants without ET. This dampened excitability manifested specifically in variables necessitating heightened recruitment and synchronization of motor neurons, as observed in the input-output (I/O) curve and motor evoked potentials (MEPs) amplitude during muscle contraction. Interestingly, an increase in cortical silent period (CSP) duration was observed in the right hemisphere at 4 and 6 hours post-intake. Though this is consistent with the diminished excitability of the right motor cortex, the temporal disparity suggests a potentially distinct pathway affected by propranolol.

Conversely, individuals with ET demonstrated heightened excitability in the right cortex at 4 hours after propranolol administration, a response that mostly endured at the 6-hour mark. Simultaneously, reduced excitability was discernible in the left cortex of ET subjects, an outcome not observed in the non-ET sample group. Furthermore, alterations in motor threshold manifested exclusively in the ET sample group’s right hemisphere.

Our findings imply the simultaneous activation and inhibition of multiple pathways contingent upon the individual’s activation state, particularly the motor threshold. Motor thresholds have been associated with voltage-gated sodium channels, while I/O curve and CSP dynamics are regulated by GABAergic signaling [31]. Moreover, glutamatergic signaling modulates the I/O curve. Notably, propranolol, through adrenergic modulation, can influence both GABAergic and glutamatergic signaling, in addition to possibly affecting sodium channels through distinct mechanisms [31].

Regarding the disparate hemispheric response to propranolol, prior research has also demonstrated its capacity to differentially attenuate the excitability of specific central nervous structures. For instance, a study investigating emotional responses to facial expressions revealed propranolol’s impact on the left basolateral amygdala [40]. Similarly, research exploring rostral anterior cingulate cortex responses in chronic PTSD patients reported increased right ACC activation following propranolol administration [23]. Moreover, data from an autism spectrum disorder study suggested propranolol’s hemisphere-specific effect [24]. In this context, transcallosal signaling holds particular significance given that the glutamatergic transcallosal pathways, connecting with pyramidal tract neurons through GABAergic interneurons, are implicated in the modulation of motor cortex inhibition and facilitation [41]. Transcallosal inhibition, induced by voluntary contralateral hand movement, has been observed to influence tremor intensity in individuals with essential tremor [42]. Additionally, prolonged administration of propranolol has been reported to increase GABA content, synthesis, and turnover rate, while also regulating the expression of the glutamate surface receptor GluA1 in various CNS structures such as the hypothalamus, pons, and amygdala [43,44]. From this, we can hypothesize that propranolol might modulate signaling within the transcallosal pathway, thereby potentially altering motor cortex excitability in a differential manner.

The distinctive impact of propranolol on ET subjects compared to non-ET subjects lends credibility to other potential mechanisms:

1. Subcortical Circuitry: Propranolol might mitigate essential tremor’s over-activation of the direct pathway within the basal ganglia-thalamus circuitry, as evidenced by decreased glucose metabolism in the left basal ganglia among propranolol responders [45]. However, due to limited sample size, the responder/non-responder comparison could not be explored.
2. Hemispheric Activity and Compensation: Healthy subjects’ right M1 activity has been linked to sympathetic activity, while left M1 activity is inversely related. Although our first experiment supports this hypothesis, the lack of left hemisphere effect might be attributed to hemispheric dominance or compensatory mechanisms [46]. Further studies with left-handed subjects and paired-pulse TMS are warranted.
3. ET-Driven Adaptations: ET’s chronic nature and converging central oscillators may lead to a subclinical hyper-excitation point, rendering it amenable to pharmacological interventions. This assumes significance within a subtype of acute progression.

Future studies should account for peripheral adrenergic modulation and incorporate hydrophilic beta-adrenergic antagonists to distinguish central from peripheral effects. Additionally, the incorporation of TMS paired-pulse methodologies could elucidate the impact of GABAergic signaling on intracortical facilitation/inhibition, thereby directly investigating interhemispheric modulation [31].

Nonetheless, our experiments bear limitations. Given the focus on dissecting propranolol’s mechanisms in ET, specific demographic variables were not considered, necessitating future investigations incorporating responder vs. non-responder, familial history, and disease onset subgroups. While neuroimaging was unavailable to corroborate our TMS results, cardiovascular response served as a surrogate marker for beta-adrenergic antagonism, albeit considering instrument frame rate constraints. Furthermore, the sample size used in the study, both for the ET group and the control group, was relatively small. Consequently, for the complex factorial analysis described, it is likely that the statistical analysis may not have had enough power to detect more subtle differences in the variables. Hence, the results presented here should be regarded solely as an exploratory study, and their conclusions should be validated in subsequent studies with larger cohorts.

In conclusion, our study pioneers the investigation of propranolol’s influence on motor cortex in essential tremor subjects. Despite limitations, our pilot study unveils central effects and a differential hemisphere-specific impact on ET subjects, as reflected by I/O curve alterations. These effects are contingent upon hemisphere and disease presence. In non-ET individuals, propranolol diminishes right cortical excitability, while in ET subjects, it heightens right cortical excitability and diminishes left cortical excitability. Future studies encompassing interhemispheric communication in specific ET subpopulations and left-handed subjects will enrich our understanding of propranolol’s neural mechanisms in ET.

In summary, the intricate interplay between propranolol and neural dynamics presents a spectrum of potential effects, spanning from amplified input-output curves characterized by cortical disinhibition to dampened I/O curves associated with decreased neural excitability. The selective blockade of beta-adrenergic receptors may foster inhibitory interneuron activity, culminating in heightened neural response thresholds and intensified inhibitory feedback within neural circuits. Conversely, beta receptor inhibition could lead to an overall reduction in neural excitability, affecting both excitatory and inhibitory neurons and thereby achieving a more balanced diminishment of neural activity and responsiveness. The exploration of these divergent outcomes not only sheds light on the intricate mechanisms underlying propranolol’s impact on neural dynamics but also underscores the need for continued investigation to decipher the precise modulation of neural circuits by this ET treatment.

## Additional Files

The additional files for this article can be found as follows:

10.5334/tohm.829.s1 Supplementary Figure 1. Flowchart of subject recruitment for the ET and non-ET sample groups (EKG, electrocardiogram; MRI, magnetic resonance imaging).

10.5334/tohm.829.s2 Supplementary Figure 2. Cardiovascular data (heart rate, systolic blood pressure and diastolic blood pressure) for the ET and reference sample groups after placebo or propranolol intake (*p < 0.05 for the subjects administered propranolol).
