# Supplementary material for: Effect of Propranolol on Motor Cortex Excitability in Essential Tremor: An Exploratory Study
Source: Tremor Other Hyperkinet Mov (N Y). 2024 Jan 2;14:1. doi: 10.5334/tohm.829 (PMC10768567; doi:10.5334/tohm.829)

Supplementary Figure 1. Flowchart of subject recruitment for the ET and non-ET sample groups (EKG, electrocardiogram; MRI, magnetic resonance imaging).

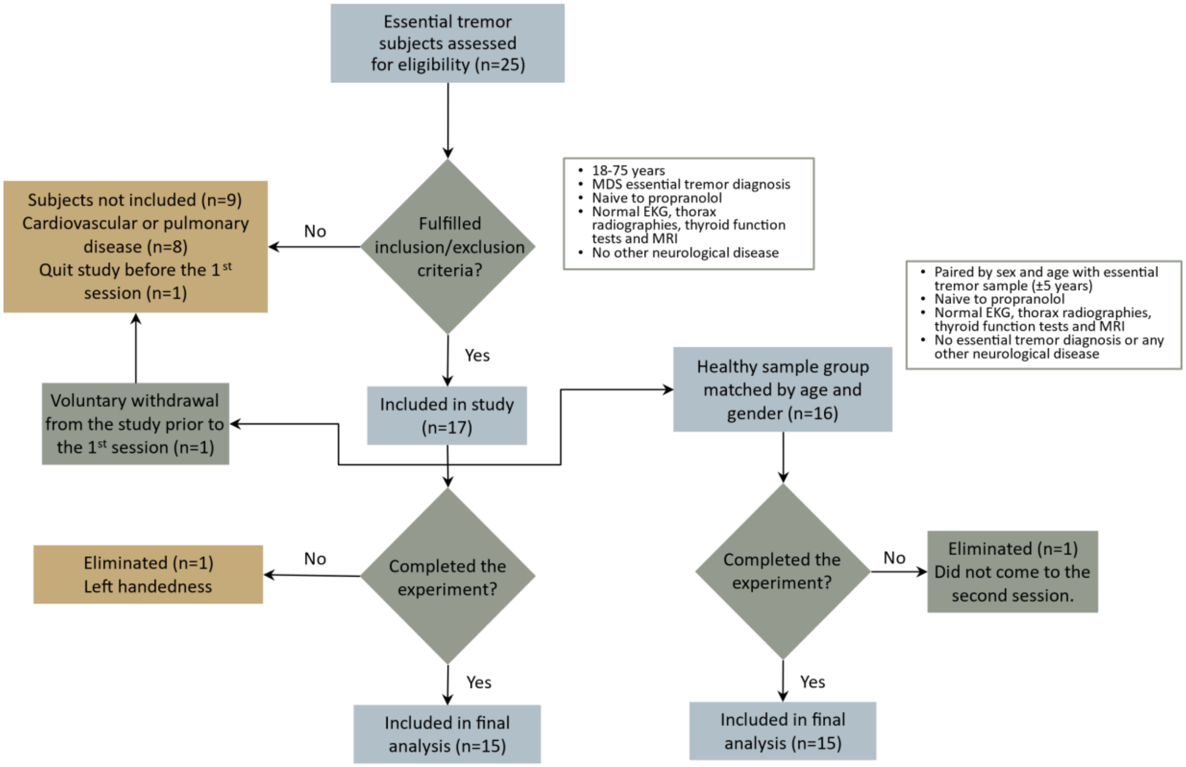

Supplement: Supplementary Figure 1. — Flowchart of subject recruitment for the ET and non-ET sample groups (EKG, electrocardiogram; MRI, magnetic resonance imaging). [file tohm-14-1-829-s1.pdf]
