# Supplementary material for: Effect of Propranolol on Motor Cortex Excitability in Essential Tremor: An Exploratory Study
Source: Tremor Other Hyperkinet Mov (N Y). 2024 Jan 2;14:1. doi: 10.5334/tohm.829 (PMC10768567; doi:10.5334/tohm.829)

Supplementary Figure 2. Cardiovascular data (heart rate, systolic blood pressure and diastolic blood pressure) for the ET and reference sample groups after placebo or propranolol intake (*$p < 0.05$ for the subjects administered propranolol).

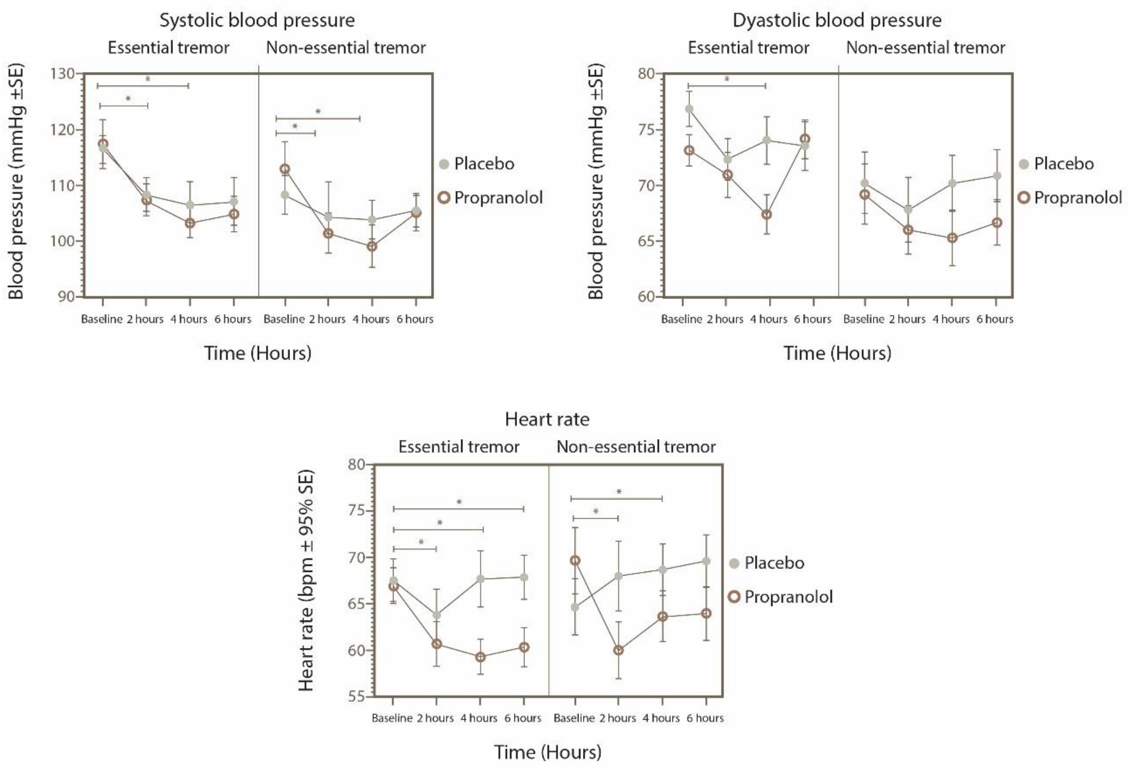

Supplement: Supplementary Figure 2. — Cardiovascular data (heart rate, systolic blood pressure and diastolic blood pressure) for the ET and reference sample groups after placebo or propranolol intake (*p < 0.05 for the subjects administered propranolol). [file tohm-14-1-829-s2.pdf]
